# A word of caution: never use tacks for mesh fixation to the diaphragm!

**DOI:** 10.1007/s00464-018-6050-2

**Published:** 2018-01-16

**Authors:** F. Köckerling, C. Schug-Pass, R. Bittner

**Affiliations:** 1 Department of Surgery and Center for Minimally Invasive Surgery, Academic Teaching Hospital of Charité Medical School, Vivantes Hospital, Neue Bergstrasse 6, 13585 Berlin, Germany; 2 Hernia Center, Winghofer Medicum, Winghofer Strasse 42, 72108 Rottenburg am Neckar, Germany

**Keywords:** Mesh fixation, Tacks, Cardiac tamponade, Hiatal hernia, Laparoscopic IPOM

## Abstract

**Background:**

The mesh fixation technique used in repair of hiatal hernias and subxiphoid ventral and incisional hernias must meet strenuous requirements. In the literature, there are reports of life-threatening complications with cardiac tamponade and a high mortality rate on using tacks. The continuing practice of tack deployment for mesh fixation to the diaphragm and esophageal hiatus should be critically reviewed.

**Methods:**

In a systematic search of the available literature in May 2017, 23 cases of severe penetrating cardiac complications were identified. The authors became aware of two other cases in which they acted as medical experts. Furthermore, the instructions for use issued by the manufacturers of the tacks were reviewed with regard to their deployment in the diaphragm.

**Results:**

Twenty-three of 25 cases (92%) with severe cardiac injuries and subsequent cardiac tamponade were triggered by the use of tacks in the diaphragm. In six cases (24%), these related to ventral and incisional hernias with extension to the subxiphoid area, and in 19 cases (76%) to mesh-augmented hiatoplasty. Twelve of 25 (48%) patients died as a result of pericardial and/or heart muscle injury with cardiac tamponade despite heart surgery intervention. In the tack manufacturers’ instructions for use, their deployment in the diaphragm, in particular in the vicinity of the heart, is contraindicated. Likewise, the existing guidelines urgently advise against the use of tacks in the diaphragm, recommending instead alternative fixation techniques.

**Conclusions:**

Tacks should not be used for mesh fixation in the diaphragm above the costal arch.

## Background

Compared to the open approach, laparoscopic intraperitoneal onlay mesh (IPOM) repair of ventral and incisional hernias has a lower rate of wound infections and similar recurrence and postoperative pain rates [1–4]. For fixation of the mesh in laparoscopic IPOM, the use of sutures alone or a combination with tacks is recommended [1]. In addition to other factors reported in the literature, larger defects greater than 10 cm in diameter and subxiphoid location are identified as increasing the complexity of laparoscopic IPOM in ventral and incisional hernia repair [1, 4]. In 2010, the first report of cardiac tamponade due to tack fixation of the mesh to the diaphragm in incisional hernia repair was published [5].

In hiatal hernia repair, the use of a mesh for reinforcement of large hiatal hernias leads to decreased short-term recurrence rates [6]. In a survey of the Society of American Gastrointestinal Endoscopic Surgeons (SAGES) members, 261 surgeons reported a total of 5486 hiatal hernia repairs with mesh [7].

The Society of American Gastrointestinal Endoscopic Surgeons (SAGES) guidelines for the management of hiatal hernia state that there exists inadequate evidence for a recommendation to be made regarding optimal fixation techniques [6].

In 2000, Kemppainen et al. [8] reported the first case of fatal cardiac tamponade after emergency tension-free mesh repair of a large paraesophageal hernia. The mesh was fixed with tacks.

In 2012, Frantzides et al. [9] presented a review of the literature and search of the US Food and Drug Administration’s Manufacturer and User Device Experience (MAUDE) database. They reported a total of 10 cases of cardiac tamponade in hiatal hernia repair, of which six resulted in patient mortality, and five cases of ventral hernia repair, of which four had a fatal outcome. Ten cases were caused by the helical tacker, two by sutures, one by the straight stapler, and in one case the cause was not identified [9].

All cases of severe penetrating cardiac complications known to be triggered by surgery are now described in detail below. In addition to the cases presented in the review by Frantzides et al. [9], ten further cases are included. As a consequence of these cases, numerous statements and recommendations have in the meantime been incorporated into guidelines and publications for avoidance of this severe complication. These are presented in Discussion section as a management recommendation for surgeons. The instructions for use given by the manufacturers of the mesh fixation systems are also reviewed in that context.

## Methods

In May 2017, a systematic search of the available literature was performed using Medline, PubMed, the Cochrane Library, as well as a search of relevant journals and reference lists. The following search terms were used: mesh fixation A*N*D diaphragm, mesh fixation A*N*D hiatal hernia, mesh fixation A*N*D subxiphoid hernia, mesh fixation A*N*D cardiac complications, mesh fixation A*N*D cardiac tamponade, mesh fixation A*N*D hemopericardium, tacks A*N*D cardiac complications, and mesh A*N*D cardiac tamponade. The search identified 19 relevant articles.

## Results (Table 1)

### Case 1 [10]

A 66-year-old female patient underwent surgery because of a large paraesophageal hiatal hernia. Just prior to the fundal wrap of the Nissen fundoplication, the patient suddenly became tachycardic and profoundly hypotensive. Emergency sternotomy revealed hemopericardium and a 2-cm laceration of the diaphragmatic surface of the right ventricle, which was controlled by placement of several sutures. The authors believed that the injury to the heart was secondary to a perforating needle on the contracting ventricle. The patient could be discharged from hospital on postoperative day 21.

**Table 1:** Overview of all cases with cardiac tamponade/injuries caused by hernia surgery

Case	Author	Access	Indication	Procedure	Cause	Outcome
1	Farlo et al. [10]	Laparoscopic	Large paraesophageal hernia	Nissen fundoplication	Suture	Recovery
2	Trastek et al. [11]	Thoracotomy	Gastroesophageal reflux disease	Collis-Nissen fundoplication	Suture	Death
3	Kemppainen et al. [8]	Laparoscopic	Large paraesophageal hernia with gastric incarceration	Mesh-augmented hiatoplasty	Straight hernia stapler	Death
4	Thijssens et al. 2002 [12]	Laparoscopic	Upside-down stomach	Mesh-augmented hiatoplasty	Helical tacker	Recovery
5	Müller-Stich et al. [13]	Open	Recurrent reflux disease after fundoplication	Mesh-reinforced hiatoplasty and fundophrenicopexy	Suture	Recovery
6	Müller-Stich et al. [13]	Laparoscopic	Gastroesophageal reflux disease	Mesh-augmented hiatoplasty and fundophrenicopexy	Helical tacker	Death
7	Dapri et al. 2007 [14]	Laparoscopic	Congenital diaphragmatic hernia	Mesh-augmented defect closure	Helical tacker	Recovery
8	Malmstrom et al. [5]	Laparoscopic	Incisional hernia	Intraperitoneal onlay mesh repair	Helical tacker	Recovery
9	Frantzides et al. [9]	Laparoscopic	Incisional hernia	Intraperitoneal onlay mesh repair	Helical tacker	Death
10	Frantzides et al. [9]	Laparoscopic	Unknown	Mesh-augmented hiatoplasty	Helical tacker	Death
11	Frantzides et al. [9]	Unknown	Ventral hernia	Ventral hernia repair	Helical tacker	Death
12	Frantzides et al. [9]	Unknown	Ventral hernia	Ventral hernia repair	Helical tacker	Death
13	Frantzides et al. [9]	Unknown	Ventral hernia	Ventral hernia repair	Helical tacker	Death
14	Frantzides et al. [9]	Unknown	Hiatal hernia	Hiatal hernia repair	Helical tacker	Death
15	Frantzides et al. [9]	Unknown	Hiatal hernia	Hiatal hernia repair	Helical tacker	Death
16	Frantzides et al. [9]	Unknown	Hiatal hernia	Hiatal hernia repair	Helical tacker	Recovery
17	Paz et al. [15]	Laparoscopic	Gastroesophageal reflux disease	Mesh-augmented hiatoplasty and Toupet fundoplication	Tacker	Recovery
18	Makarewicz et al. [16]	Laparoscopic	Gastroesophageal reflux disease	Mesh-augmented hiatoplasty and Nissen fundoplication	Helical tacker	Recovery
19	Jorgensen et al. [17]	Laparoscopic	Unknown	Mesh-augmented hiatoplasty	Tacker	Death
20	del Carmen Fernandez et al. [18]	Laparoscopic	Gastroesophageal reflux disease	Mesh-augmented hiatoplasty and Nissen fundoplication	Absorbable tacker	Recovery
21	del Carmen Fernandez et al. [18]	Laparoscopic	Gastroesophageal reflux disease	Mesh-augmented hiatoplasty and Nissen fundoplication	Absorbable tacker	Recovery
22	del Carmen Fernandez et al. [18]	Laparoscopic	Upside-down stomach	Mesh-augmented hiatoplasty and Nissen fundoplication	Absorbable tacker	Recovery
23	McClellan et al. (19)	Laparoscopic	Bariatric redo-procedure with large hiatal defect	Mesh closure of a large hiatal hernia defect	Absorbable tacker	Recovery
24	We are aware of an additional unreported, unpublished case, in which one the authors (F. K.) acted as medical expert	Open	Incisional hernia extending from suprapubic to subxiphoid	Intraperitoneal onlay mesh repair	Absorbable tacker	Death
25	We are aware of an additional unreported, unpublished case, in which one of the authors (R. B.) acted as medical expert	Laparoscopic	Upside-down stomach	Mesh-augmented hiatoplasty	EMS Stapler	Death

### Case 2 [11]

A 40-year-old man underwent Collis-Nissen repair of gastroesophageal reflux disease. The night of the operation he became hypotensive and was believed to have cardiac tamponade. Bedside pericardiocentesis was performed with temporary improvement. On returning to the operating room, he coded and died on the operating table. The autopsy findings showed laceration of a coronary vessel.

### Case 3 [8]

A patient with thoracic herniation and incarceration of the stomach underwent laparoscopic operation, including reduction of an intrathoracic stomach, hernia sac removal, and tension-free repair of the hiatus with polytetrafluoroethylene mesh. The mesh was fixed with a straight hernia stapler. Postoperatively, the patient developed fatal cardiac tamponade secondary to coronary vein laceration due to fixation of the mesh with a stapler.

### Case 4 [12]

An 84-year-old woman suffered from a type IV diaphragmatic hernia with a tortuous intrathoracic stomach and grade II esophagitis. The stomach was reduced into the abdominal cavity and the hernia sac resected. A coated polyester mesh was attached to the crura with 5-mm titanium helical tacks. After an uneventful recovery, the patient suddenly developed cardiogenic shock on postoperative day 14. A left thoracotomy was subsequently performed. There was active bleeding of the epicardium from a number of pointed lesions, just opposite the tackers which had penetrated the pericardium. The offending tacks were removed and the defects in the epicardium sutured. The patient was discharged 18 days after thoracotomy.

### Case 5 [13]

A 74-year-old woman presented with a request for a second antireflux surgery seven years after the primary surgery. Because of the previous surgical intervention, an upper median laparotomy was used. Mesh-reinforced hiatoplasty with anterior fundophrenicopexy was performed as an antireflux procedure. A 6 × 6 cm prolene mesh was applied and fixed to the diaphragm with 10 helical staples. A few hours postoperatively, a decrease in blood pressure occurred and the subsequent computed tomography (CT) scan confirmed suspected cardiac tamponade. An emergency relaparotomy with pericardial fenestration was performed. Venous bleeding from the myocardium of the right ventricle, probably caused by a fundophrenicopexy suture, was stopped by resuturing, the pericardium was left open, and the left hemithorax was drained. The patient could be discharged on postoperative day 24.

### Case 6 [13]

An 82-year-old man presented with an 8-cm mixed hiatal hernia with grade IV esophagitis. Surgical treatment consisted of laparoscopically mesh-augmented hiatoplasty with anterior fundophrenicopexy. Because of accidental rupture of the right crus of the diaphragm, a 15 × 15 cm prolene mesh was necessary to reconstruct the hiatus. The mesh was fixed by 16 helical tacks. During the first 24 postoperative hours, deteriorating oxygen saturation with increasing dyspnea was most notable. After 48 h, the patient experienced severe retrosternal pressure with simultaneous tachyarrhythmia and consecutive circulatory failure. The patient died despite resuscitation attempts. The autopsy revealed cardiac tamponade originating from an epicardial vascular erosion caused by one of the helical tacks.

### Case 7 [14]

A 57-year-old woman presented with right, medial, and left congenital diaphragmatic hernia defect. The hernia defect was closed laparoscopically by a running suture and then a mesh placed as an onlay fixed with helical hernia tacks. The patient developed cardiac tamponade on the second day due to tack-induced bleeding of an epicardial artery of the inferior surface of the heart, which required emergency pericardial drainage. The outcome was good, and discharge home was allowed after 1 month.

### Case 8 [5]

A 25-year-old obese patient developed a large incisional hernia after gallbladder removal with lesion of the common bile duct and open transverse revision. This was repaired laparoscopically using two meshes measuring 30 × 30 and 20 × 30 cm, respectively, sewn together. The facial defect was in a transverse incision under both rib curvatures, measuring 35 × 15 cm. The mesh was positioned ensuring an overlap of about 8 cm. The mesh was fixed using titanium spiral tackers along the margin of the mesh, with a distance of 1 cm between each tacker. On postoperative day 9, the patient exhibited a decreased state of consciousness, blood pressure of 80/50 mmHg, a heart rate of 70 beats/min, and spontaneous breathing with 40 respirations/min.

Extensive pericardial tamponade diagnosed on echocardiography was treated by percutaneous drainage, evacuating 500 ml of blood. Sternotomy showed a spiral tacker protruding through the pericardium, resulting in a lesion of 2 × 2 mm in the right ventricle. Furthermore, five spiral tackers were visible from the inside of the pericardium and two had gone through the diaphragm and into the pleural cavity. The patient was discharged a week later in good health.

### Cases 9 and 10 [9]

The authors were aware of two additional unreported, unpublished cases with cardiac injury, of which one was sustained during ventral herniorrhaphy and one during hiatal hernia repair. The senior author acted as a medical expert in these cases.

Both heart injuries were the result of helical tacks being used for fixation of the mesh to the central tendon of the diaphragm. Both patients died as a result of hemopericardium tamponade. In each of these cases, the diagnosis of cardiac tamponade was established only at autopsy.

### Cases 11 to 16 [9]

The MAUDE database (US Food and Drug Administration’s Manufacturer and User Facility Device Experience) search revealed a total of six cases of cardiac tamponade associated with the helical tacker during mesh fixation to the diaphragm. Three of these cases occurred during ventral hernia repair and all were fatal. The other three cases occurred during hiatal hernia repair and two were fatal.

### Case 17 [15]

A 61-year-old female with a history of gastroesophageal reflux disease and hiatal hernia developed hemopericardium and tamponade one day after laparoscopic hiatal hernia repair and Toupet fundoplication. The patient underwent emergency pericardiocentesis and subsequent surgical pericardial window. During surgery, a tack that had been used to secure the mesh to the inferior aspect of the diaphragm was found to have penetrated the pericardium near the right ventricle. The patient recovered without further complications.

### Case 18 [16]

A 69-year-old patient presented with gastroesophageal reflux disease and a stage III paraesophageal hernia. Nissen fundoplication was performed. For hiatoplasty a mesh was used, which was fixed to the diaphragm with six helical tackers. On postoperative day 3, the patient collapsed with signs of acute heart failure and low output syndrome secondary to cardiac tamponade. On emergency sternotomy, approximately 1500 mm^3^ of hemolyzed blood with clots was retrieved from pericardial sac. Two tacks (3 cm medially to the inferior vena cava) penetrating into the pericardial sac were noted. The patient survived this severe complication.

### Case 19 [17]

A 79-year-old woman underwent laparoscopic hiatal hernia repair with mesh implantation. Tacks were used to secure the mesh to the diaphragm. Nine hours after surgery the patient died from circulatory collapse. Autopsy showed perforation of the pericardium and the right coronary artery by a tacker.

### Case 20 [18]

A 46-year-old man with gastroesophageal reflux disease underwent Nissen fundoplication with hiatal mesh reinforcement using absorbable tacks. Forty-eight hours after surgery, the patient developed hypotension and sweating. Cardiac magnetic resonance imaging showed a large pericardial effusion. Pericardiocentesis was performed with removal of 650 cc of bloody fluid. The pericardial drain could be removed two days later. The patient’s further course was uneventful.

### Case 21 [18]

A 62-year-old woman with gastroesophageal reflux disease and a large hiatal hernia underwent Nissen fundoplication with mesh hiatoplasty using absorbable tacks. On postoperative day 5, the patient developed acute respiratory distress syndrome with hypotension and oliguria. A thoracic CT scan showed a bilateral pleural effusion and a large pericardial effusion that caused the collapse of the right atrium and ventricle. She was taken back to the operating room for urgent surgical pericardial drainage. After that, the postoperative course was uneventful.

### Case 22 [18]

A 70-year-old woman presented with a giant hiatal hernia with intrathoracic stomach. The patient underwent laparoscopic Nissen fundoplication with hiatal mesh reinforcement using absorbable tacks. On postoperative day 2, the patient developed hypotension, tachycardia, and irregular pulse.

A CT scan revealed an extrapericardial hematoma in the hernia sac with mild left atrium compromise. Conservative treatment was decided and the patient discharged on postoperative day 15.

### Case 23 [19]

During a redo bariatric procedure, a hiatal defect of 10 cm could not be initially repaired due to its significant size, rigidity of the lateral edges, and the anterior location, and thus mesh repair was performed. Due to a difficult suturing angle of the most posterior area of the defect, the mesh was secured to the anterior portion of the right and left crus with several absorbable tacks. On postoperative day 1, the patient became tachycardic and the echocardiogram revealed a moderate-sized pericardial effusion with a partially filled right atrium and partially collapsed right ventricle consistent with early tamponade. During percutaneous pericardiocentesis, 370 ml of bloody fluid was aspirated with subsequent immediate normalization of vital signs and relief of symptoms. The patient was discharged home two days later.

### Case 24

The authors are aware of an additional unreported, unpublished case with cardiac injury during open incisional hernia repair in IPOM technique. The first author (F. K.) acted as medical expert in that case. This related to a 52-year-old male patient with a large incisional hernia and status post multiple laparotomies following emergency left hemicolectomy, Hartmann’s operation, and Hartmann’s reversal operation. Open IPOM operation with placement of a 50 × 30 cm polypropylene mesh was performed. Since the incisional hernia extended from the lower abdomen to the subxiphoid area, the mesh was positioned from the symphysis to the subdiaphragmatic area. The mesh was fixed with sutures and reinforced with absorbable tackers between the sutures. Postoperatively, the patient experienced increasing thoracic pain. On postoperative day 1, a suspected heart attack was diagnosed and treatment initiated with platelet aggregation inhibitors. Due to increasing circulatory instability, the patient was transferred to the cardiac surgery clinic for treatment on postoperative day 2. Cardiac tamponade was diagnosed in the cardiac surgery clinic and initially treated by means of emergency pericardial drainage. Despite that, emergency sternotomy had to be performed and the patient died on the operating table. Autopsy revealed four tacks in the vicinity of the diaphragm which had penetrated the pericardium. One tack had perforated the right ventricle causing bleeding into the pericardium and cardiac tamponade.

### Case 25

The authors are aware of an additional unreported, unpublished case with cardiac injury during mesh-augmented hiatoplasty of an upside-down stomach. The senior author (R. B.) acted as medical expert in this case. In the 65-years-old female patient the mesh was partially fixed with sutures and EMS staples. A branch of a single EMS staple did not close, perforated the pericardium and injured a vene at the anterior part of the heart. Subsequently, a cardiac tamponade developed, which was not diagnosed in time. The patient died on postoperative day 3.

### Instructions for use by the manufacturers of the tacks

The instructions for use for the most commonly employed tacks contain the following contraindications:

#### ProTack (Medtronic—Covidien)

This device should not be used in tissues that have direct anatomical relationship to major vascular structures. This would include the deployment of helical fasteners in the diaphragm in the vicinity of the pericardium, aorta, or inferior vena cava during diaphragmatic hernia repair.

#### AbsorbaTack (Medtronic—Covidien)

This device should not be used in tissues that have a direct anatomic relationship to major vascular structures. This would include deployment of tacks in the diaphragm in the vicinity of the pericardium, aorta, and inferior vena cava during diaphragmatic hernia repair.

#### SecureStrap (Ethicon)

This device should not be used in tissues that have a direct anatomic relationship to major vascular structures. This would include the deployment of fasteners in the diaphragm in the vicinity of the pericardium, aorta, or inferior vena cava during diaphragmatic hernia repair.

#### SorbaFix (Bard)

Carefully inspect the area in the vicinity of the tissue being fastened to avoid inadvertent penetration of underlying structures such as nerves, vessels, viscera, or bone. Use of the SorbaFix in the close vicinity of such underlying structures is contraindicated.

## Discussion

Twenty-five cases of life-threatening complications due to mechanical injuries to the pericardium and heart during hernia surgery are known from the literature and expert witness processes. In three cases (12%), the pericardium or heart injury was caused by sutures and in 22 cases (88%) by the use of tacks. The clinical implications of such injuries were generally cardiac tamponade necessitating pericardial drainage and/or open heart surgery with sternotomy. The mortality rate associated with this severe complication is 48%. The underlying indications for surgery were ventral and incisional hernia repair in six cases (24%) and mesh-augmented hiatoplasty in 19 cases (76%). The tacks used included both non-absorbable titanium and absorbable types. The majority of tack-mediated injuries were caused by mesh fixation to the diaphragm or diaphragmatic crura. Mesh fixation in this critical anatomic region is needed in a variety of situations ranging from mesh-augmented hiatoplasty for treatment of gastroesophageal reflux disease (GERD) through paraesophageal hernias to upside-down stomach and ventral and incisional hernias with extension to the subxiphoid area.

The severe complications described here in association with the use of tacks in the vicinity of the diaphragm and esophageal hiatus naturally raise the question as to whether tacks should at all be used in this region. The thickness of the diaphragm muscle ranges from 1.5 to 5.4 mm [9]. The central tendon of the diaphragm averages only 2.9–3.00 mm in thickness [9]. The depth of penetration, not including the thickness of the mesh, of the various tacks on the market ranges between 3.7 and 5.9 mm. The tack manufacturers’ instructions for use contraindicate the deployment of tacks in the vicinity of the heart, major vascular structures, and diaphragm.

The SAGES guidelines for laparoscopic ventral hernia repair state that fixation of meshes above the costal margins should not be performed with sutures placed between the ribs [4]. Furthermore, while fixation with tacks may by feasible, it should generally be avoided to prevent lung or cardiac injury or injuries to the neurovascular bundles running along the inferior surface of each rib [4].

The guidelines for laparoscopic treatment of ventral and incisional abdominal wall hernias of the International Endohernia Society (IEHS) recommend not to fix the proximal part of the mesh for subxiphoid hernia repair [1].

The SAGES guidelines for management of hiatal hernia state that care should be taken about the mesh fixation technique. Particularly tacks can breach the aorta or pericardium when applied low on the left crus or anteriorly near the apex of the crura.

As an alternative, Rodriguez et al. [20] advocate for extreme care when suturing mesh in hiatal hernia repair at the diaphragm, taking very superficial bites just to encourage scarring to the diaphragm and avoiding fixation by tacks at all costs.

Poris et al. [21] reported on laparoscopic repair of a subxiphoid hernia. Because of the location of the defect and the proximity to the patient’s heart and diaphragm, they believed that tacks and transfascial sutures could not be safely placed and they therefore allowed for additional overlap of 8 cm superior to the uppermost aspect of the defect. Upon desufflation of the abdomen, the liver was noted to buttress the mesh, holding it in place.

The IEHS guidelines also recommend that mesh overlap should be sufficient, especially in the proximal retro-xiphoid space [1]. Von Rahden et al. [22] recommended for mesh fixation to the diaphragm in subxiphoid hernia repair the use of fibrin glue in the proximal third.

The SAGES guidelines propose, as an alternative and safer option to prosthetic fixation, above the costal margin in subxiphoid hernia repair, allowing the mesh to drape over the diaphragm superiorly without fixation, and full-thickness fixation to the edge of the costal margin and xiphoid process away from the edge of the prosthetic [4].

In conclusion, no tacks should be used for mesh fixation in surgical repair of hiatal hernias and of ventral and incisional hernias with extension to the subxiphoid area. In the tack manufacturers’ instructions for use, the deployment of tacks for mesh fixation in the vicinity of the diaphragm is contraindicated. Likewise, the guidelines of the surgical societies advise against the use of tacks in this critical anatomic region. The cases of severe complications and very high mortality rate presented here more than adequately prove that the use of tacks in the diaphragm is too dangerous and should therefore be absolutely avoided. Alternative approaches that present no risk to patients are shown to be sufficiently effective. One could argue this is just case report series, but an important message unlikely to be reported often and as such should be published.
